# Thiouracil and triazole conjugate induces autophagy through the downregulation of Wnt/β‐catenin signaling pathway in human breast cancer cells

**DOI:** 10.1002/iub.2917

**Published:** 2024-09-10

**Authors:** Bada Yoon, Basappa Basappa, Shreeja Basappa, Omantheswara Nagaraju, Mahendra Madegowda, K. S. Rangappa, Gautam Sethi, Kwang Seok Ahn

**Affiliations:** ^1^ Department of Science in Korean Medicine Kyung Hee University Seoul Korea; ^2^ Laboratory of Chemical Biology, Department of Studies in Organic Chemistry University of Mysore Mysore Karnataka India; ^3^ Department of Chemistry BITS‐Pilani Hyderabad Campus Hyderabad India; ^4^ Department of Studies in Physics University of Mysore Mysore Karnataka India; ^5^ Department of Pharmacology, Yong Loo Lin School of Medicine National University of Singapore Singapore Singapore

**Keywords:** autophagy, breast cancer, TTP‐8, Wnt/β‐catenin pathway

## Abstract

Autophagy is vital for maintaining cellular homeostasis by breaking down unnecessary organelles and proteins within cells. Its activity varies abnormally in several diseases, including cancer, making it a potential target for therapeutic strategies. The Wnt/β‐catenin signaling pathway significantly impacts cancer by stabilizing β‐catenin protein and promoting the transcription of its target genes. Therefore, we aimed to identify candidate substances targeting this signaling pathway. We designed and tested a thiouracil conjugate, discovering that TTP‐8 had anti‐tumor effects on human breast cancer cell lines MCF‐7 and MDA‐MB231. Our findings showed that TTP‐8 upregulated the expression of LC3 protein, a marker of autophagy in breast cancer cells, suggesting that TTP‐8 might induce autophagy. Further analysis confirmed an increase in autophagy‐related proteins, with consistent results obtained from flow cytometry and confocal microscopy. Interestingly, the induction of LC3 expression by TTP‐8 was even more pronounced in MCF‐7 and MDA‐MB231 cells transfected with β‐catenin siRNA. Thus, our research supports the idea that the Wnt/β‐catenin signaling pathway influences the regulation of autophagy‐related proteins, thereby inducing autophagy. This suggests that TTP‐8 could serve as a novel agent for treating breast cancer.

## 1 INTRODUCTION

Breast cancer (BC) ranks as the second most prevalent cancer globally and is the fifth leading cause of death, with about 42,000 women succumbing to the disease annually.^1^ Factors such as hormones, genetics, age, and childbirth contribute to the risk of BC.^2^, ^3^ However, the exact pathogenesis of BC remains unclear. Common treatments include chemotherapy, radiation therapy, and surgery, which can lead to various side effects. Although advancements in medical technology and diverse treatment options have improved survival rates, the incidence of BC continues to rise annually, and survival rates can vary significantly based on the cancer type.^4^, ^5^ Thus, there is a pressing need to explore and develop new treatment options for BC.

Autophagy is a crucial intracellular process that maintains homeostasis by eliminating damaged organelles and toxic misfolded proteins under stress conditions.^6^, ^7^ This process is implicated in various diseases, including cancer, obesity, diabetes, and neurodegenerative disorders, when dysregulated. Autophagy begins with the nucleation of a phagophore, which expands into an autophagosome that degrades these substances.^8^, ^9^ The production of autophagosomes is regulated and conserved across evolution by autophagy‐related (ATG) genes. Proteins crucial for the maturation and elongation of autophagosomes, such as Beclin‐1, are involved, with elongation further regulated by ATGs. Pro‐LC3 is transformed into LC3 I by the ATG complex and subsequently into LC3 II through interaction with phosphatidylethanolamine (PE) and ATG7.^10^, ^11^ Mature autophagosomes then fuse with lysosomes to form autolysosomes, which selectively remove degradable material.^12^ The protein p62, serving as an autophagic flux marker, is integral to the autophagy process by binding to and directing unnecessary proteins to the autophagosome for degradation. Additionally, p62 is crucial as it interacts with other autophagy‐related proteins, influencing both the activation and inhibition of autophagy.^13^, ^14^, ^15^

The Wnt/β‐catenin signaling pathway regulates crucial physiological functions such as cell survival, proliferation, and metastasis, but its dysregulation is linked to several severe diseases, including various cancers.^16^, ^17^ Specifically, abnormal activation of β‐catenin plays a critical role in the onset and progression of many cancers, notably colon cancer.^18^ Activation of the Wnt protein occurs through the binding of the Frizzled (FZD) receptor and the lipoprotein receptor‐related protein 5/6 (LRP5/6) to the Wnt ligand. Following this interaction, the complex is disrupted, leading to the stabilization and accumulation of β‐catenin in the cytosol and its subsequent translocation to the nucleus, where it activates transcription of target genes.^18^, ^19^, ^20^ Numerous studies have highlighted the link between Wnt/β‐catenin signaling and autophagy. Consequently, targeting the downregulation of Wnt/β‐catenin signaling presents a potential strategy for chemotherapy.

**FIGURE 1:**
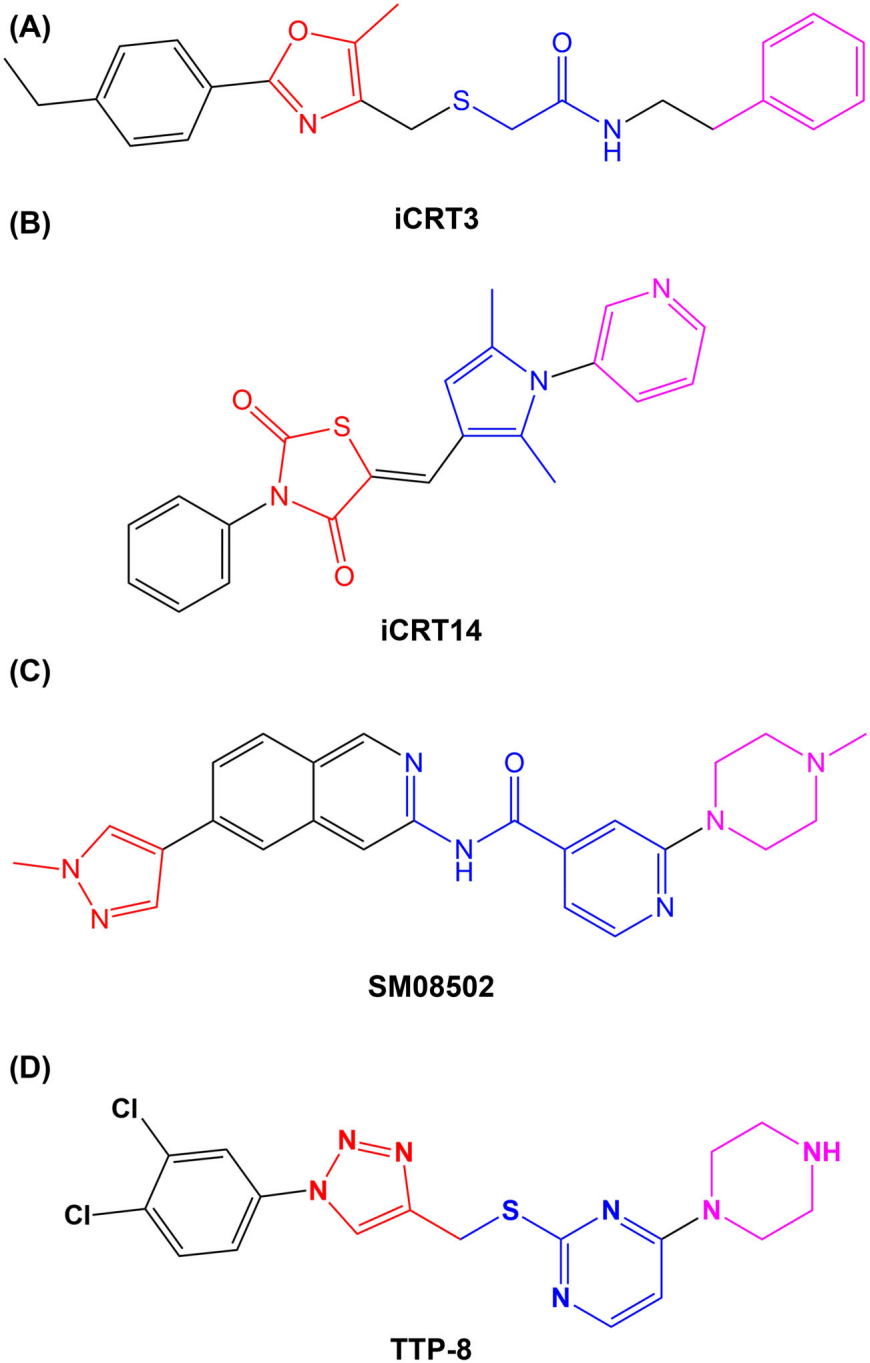
The chemical structure of TTP‐8. (A) Structure of iCRT3. (B) Structure of iCRT14. (C) Structure of SM08502. (D) Structure of TTP‐8.

iCRT3 (Figure 1A) acts as an inhibitor targeting both Wnt and β‐catenin‐responsive transcription. It significantly reduces TOP Flash activity and lowers the levels of NTSR1. The inhibitor effectively diminishes the anti‐apoptotic effects of neurotensin (NTS) and Wnt3a, largely neutralizing their action.^21^ Another compound, iCRT14, shown in Figure 1B, disrupts the interaction between TCF and DNA, as well as the binding between TCF and β‐catenin. In the case of BT‐549 cells, iCRT14 demonstrated a capacity to inhibit cell proliferation in a dose‐ and time‐dependent manner, though it was less potent than iCRT3.^22^, ^23^ Additionally, SM08502, illustrated in Figure 1C, was found to decrease Wnt signaling and gene expression by targeting the phosphorylation of the serine and arginine‐rich splicing factor and disrupting spliceosome activity in solid tumors, suggesting a role for alternative splicing mechanisms. Moreover, Wnt antagonists like Frzb/sFRP3 and Dkk1 have shown to inhibit triiodothyronine (T3) signaling, indicating a potential link between thiouracil‐induced hypothyroidism and the Wnt/beta‐catenin pathway.^24^

In this study, we developed a thiouracil and triazole conjugate, TTP‐8, and explored the type of cell death it induces in BC cells, as well as the underlying mechanisms. We observed that cell death occurred via autophagy, with the involvement of TTP‐8 confirmed through the expression of the p62 protein, a marker of autophagic flux. Additionally, we found that TTP‐8 effectively downregulates Wnt/β‐catenin signaling, facilitating autophagy in BC cells.

## 2 MATERIALS AND METHODS

### 2.1 Reagents

The compound 2‐(((1‐(3,4‐dichlorophenyl)‐1H‐1,2,3‐triazol‐4‐yl)methyl)thio)‐4‐(piperazin‐1‐yl)pyrimidine (TTP‐8) was synthesized and its chemical properties and initial anti‐cancer effects were described recently.^25^ The TTP‐8 stock solution was prepared in dimethyl sulfoxide (DMSO) at a concentration of 50 mM and stored at −20°C. RPMI‐1640 medium, fetal bovine serum (FBS), and Trypsin/EDTA buffer were purchased from Thermo Scientific HyClone (Waltham, MA, USA). 3‐(4,5‐Dimethylthiazol‐2‐yl)‐2,5‐diphenyltetrazolium bromide (MTT), sodium dodecyl sulfate (SDS), glycine, Tris base, NaCl, isopropanol, sodium chloride, monodansylcadaverine (MDC), HCl, bovine serum albumin (BSA) and 3‐methyladenine (3‐MA) were obtained from Sigma‐Aldrich (St. Louis, MO, USA). Acridine orange (AO) was purchased from ImmunoChemistry Technologies (Bloomington, Minnesota, USA). Alexa Fluor® 488 donkey anti‐rabbit IgG (H + L) antibody and Alexa Fluor™ 594 donkey anti‐rabbit IgG (H + L) antibody was purchased from Invitrogen (Eugene, Oregon, USA). The Neon™ Transfection System was purchased from Invitrogen (Carlsbad, CA, USA). Antibodies against LC3, Atg7, p‐Beclin‐1, Beclin‐1, p62, β‐catenin antibodies were procured from Cell Signaling Technology (Massachusetts, USA). Antibodies against Wnt3a, FZD‐1, p‐GSK3β (Ser9), p‐GSK3β (Tyr216), GSKβ, β‐TrCP, α‐tubulin, and Lamin B, β‐actin antibodies, β‐catenin siRNA, and scrambled siRNA were purchased from Santa Cruz Biotechnology (Santa Cruz, Dallas, TX, USA).

### 2.2 Cell lines and culture conditions

Human BC cell lines (MCF‐7 and MDA‐MB231) were purchased from Korean Cell Line Bank (Seoul, Korea). Human breast epithelial MCF‐10A cell line was procured from American Type Culture Collection (ATCC). MCF‐7 and MDA‐MB231 cells were cultured in RPMI1640 medium and MCF‐10A cells cultured in DMEM/F‐12 medium. All the medium was supplemented with 10% fetal bovine serum (FBS) and 1% penicillin–streptomycin. These cells were maintained at 37°C under 5% CO_2_ conditions.

### 2.3 MTT assay

Cytotoxicity was analyzed by the MTT assay. MCF‐7, MDA‐MB231, and MCF‐10A cells (1 × 10^4^ cells/well) were seeded in 96 plates. Following overnight incubation, various concentrations of TTP‐8 (0, 2.5, 5, 10, 20, 30 μM) were administered for 24 h. Subsequently, 2 mg/mL of MTT solution was added to each well containing the treated cells and incubated for 2 h. The supernatant was then removed, and DMSO was added and allowed to incubate for another 2 h at 37°C. The formation of formazans was measured at 570 nm using a VARIOSKAN LUX spectrophotometer (Thermo Fisher Scientific Inc., Waltham, MA). Cell viability was expressed as a percentage relative to the untreated control cells.^26^, ^27^

### 2.4 Western blot analysis

MCF‐7 and MDA‐MB231 (1 × 10^4^ cells/well) cells were seeded on 6 well plate and incubated under specified conditions. Subsequently, Western blot analysis was conducted to detect specific proteins.^28^ Whole cell lysates were prepared, and protein concentrations were determined using the Bradford assay. The proteins were separated by sodium dodecyl sulfate polyacrylamide gel electrophoresis (SDS‐PAGE) and transferred onto nitrocellulose membranes. These membranes were then blocked with 5% skim milk for 1 h. Overnight incubation with primary antibodies was performed at a dilution of 1:3000, followed by a 1 h incubation with secondary antibodies at a dilution of 1:5000 the next day.^29^ The membranes were then detected using enhanced chemiluminescence (EZ‐Western Lumi Femto, DOGEN).

### 2.5 AO staining analysis using flow cytometry

MCF‐7 and MDA‐MB231 cells (5 × 10^5^ cells/well) were seeded in 6 plates and then TTP‐8 (30 μM) or 3‐MA (2 mM) was treated. Cells were harvested with trypsin/EDTA and washed with PBS. 1.9 μL of AO staining buffer per sample was treated in 500 μL PBS and incubated at 37°C for 30 min. After staining, the samples were analyzed using BD Accuri™ C6 Plus Flow Cytometer (BD Biosciences, Becton‐Dickinson, Franklin Lakes, NJ) with BD Accuri C6 Plus software.^7^

### 2.6 MDC staining

MCF‐7 and MDA‐MB231 cells (2 × 10^4^ cells/well) were seeded in 8 well chambers. The cells were then treated with TTP‐8 (30 μM) or 3‐MA (2 mM). For staining, 0.2 μL of MDC staining buffer was added to each sample in 200 μL of PBS and incubated at 37°C for 30 min.^15^ Following incubation, cells were treated with a mounting medium (Sigma‐Aldrich) and left overnight. Visualization was carried out using a FluoView FV1000 confocal microscope (Olympus, Japan).

### 2.7 Immunocytochemistry

MCF‐7 and MDA‐MB231 cells (2 × 10^4^ cells/well) were seeded in 8 well chambers. The cells were fixed using 4% paraformaldehyde (PFA) in 1× PBS for 20 min at room temperature and subsequently rinsed with PBS.^30^, ^31^, ^32^ Cells were then permeabilized with 0.2% Triton X‐100 for 10 min at room temperature and blocked using 5% BSA for 1 h. Overnight incubation with primary antibodies diluted in 5% BSA (in PBS) was conducted at 4°C.^10^ Following this, cells were incubated with secondary antibodies, either Alexa Fluor® 488 donkey anti‐rabbit IgG (H + L) or Alexa Fluor™ 594 donkey anti‐rabbit IgG (H + L), for 1 h at room temperature. Nuclei were stained with DAPI (1 mg/mL) for 3 min and mounted using Fluorescent Mounting Medium (Golden Bridge International Labs, Mukilteo, WA). Imaging and analysis were performed using an Olympus FluoView FV1000 confocal microscope (Tokyo, Japan).^32^

### 2.8 AO staining analysis by using confocal microscope

MCF‐7 and MDA‐MB231 cells (2 × 10^4^ cells/well) were seeded in 8 well chambers. The cells were then treated with TTP‐8 (30 μM) in a time‐dependent manner. After rinsing with PBS, each sample was stained with 0.2 μL of AO staining buffer in 200 μL of PBS and incubated at 37°C for 30 min. Subsequently, the samples were covered with a mounting medium (Sigma‐Aldrich) overnight. Imaging was performed using a FluoView FV1000 confocal microscope (Olympus, Japan).

### 2.9 Transfection of β‐catenin siRNA by using neon™ transfection system

For the transfection of BC cells, we used the Neon™ Transfection System (Invitrogen, Carlsbad, CA). MCF‐7 and MDA‐MB231 cells were harvested in 10% FBS medium and resuspended in 120 μL of Neon Resuspension Buffer (R buffer) per million cells. The cells were transfected via electroporation, using 50 nM of β‐catenin small interfering RNA (siRNA) and scrambled (s.c.) siRNA, and then placed into a sterile microcentrifuge tube. Next, 2 mL of Neon Electrolytic Buffer (E buffer) was added to the Neon Pipette Station, followed by the insertion of the Neon Tip (disposable tip 100 μL) which drew up the cell mixture and Neon Resuspension Buffer R into the Neon tube. MCF‐7 cells received two pulses at 950 V with a pulse width of 40 ms, while MDA‐MB231 cells were pulsed four times at 1400 V with a pulse width of 10 ms. Following transfection, the cells were incubated for 24 h. Subsequently, TTP‐8 (30 μM) was administered for either 6 or 24 h before performing Western blot analysis.^33^

### 2.10 Immunocytochemistry (ICC) analysis

For transfecting BC cells, we employed the Neon™ Transfection System (Invitrogen, Carlsbad, CA). MCF‐7 and MDA‐MB231 cells were harvested in 10% FBS medium and resuspended in 120 μL of Neon Resuspension Buffer (R buffer) per million cells. Electroporation was used for transfection, applying 50 nM of β‐catenin small interfering RNA (siRNA) and scrambled (s.c.) siRNA. The cells were then distributed into sterile microcentrifuge tubes.^34^, ^35^ Following this, 2 mL of Neon Electrolytic Buffer (E buffer) was placed into the Neon Pipette Station, and the Neon Tip (disposable tip 100 μL) was used to aspirate the cell mixture and Neon Resuspension Buffer R into the Neon tube.^34^ For electroporation settings, MCF‐7 cells were given two pulses at 950 V with a 40 ms pulse width, whereas MDA‐MB231 cells received four pulses at 1400 V with a 10 ms pulse width. After 24 h of incubation post‐transfection, cells were detached using trypsin/EDTA and seeded at 2 × 10^4^ cells/well in 8‐well chambers. Subsequently, TTP‐8 (30 μM) was administered for 24 h, followed by ICC analysis.^15^

### 2.11 Molecular docking analysis

Utilizing AutoDock 4.2, standard docking procedures were performed to investigate the binding pose of TTP‐8 within the β‐catenin. The X‐ray crystallographic structure of the β‐catenin/Tcf complex (PDB: 1G3J) sourced from the Protein Data Bank served as the basis for molecular docking analyses. Prior to docking, the protein was prepared by eliminating the Tcf residue and crystalline water, introducing polar hydrogen atoms, and conducting residue modification and repair. A grid box with dimensions of 100 Å × 60 Å × 126 Å and a spacing of 0.80 Å was generated to encompass the entire protein, centered on the ligand's mass center. Autogrid 4 was employed to generate energy grid maps for all potential ligand atom types before docking, with subsequent analysis of docking modes conducted using Maestro (Schrödinger Inc.).^36^, ^37^

### 2.12 Statistical analysis

All the numerical values have been represented as the mean ± SD. Statistical significance of the data was evaluated using the Student unpaired *t*‐test. Significance was set at *p < .05, **p < .01, and ***p < .001(Sigma Plot 10.0, SYSTAT Software, California, USA).

## 3 RESULTS

### 3.1 TTP‐8 modulated the autophagy‐related gene products

**FIGURE 2:**
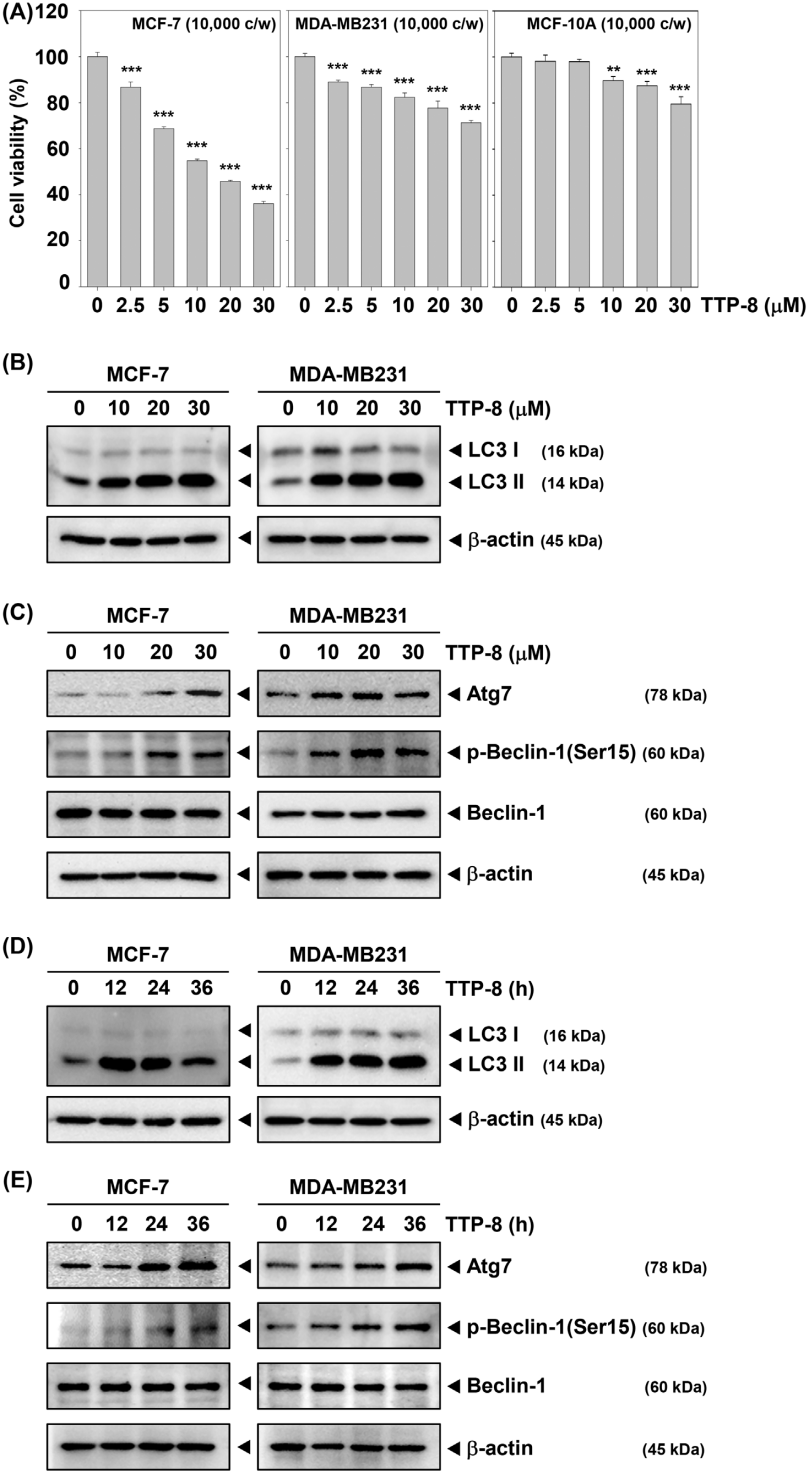
TTP‐8 regulates the expression of proteins related to autophagy in BC cells. (A) MCF‐7, MDA‐MB231, and MCF‐10A cells (1 × 10^4^ cells/well) were treated with TTP‐8 (0, 2.5, 5, 10, 20, and 30 μM) for 24 h. Then, cell viability was measured by MTT assay. Data represent means ± SD. *p < .05, **p < .01 versus non‐treated cells. (B and C) MCF‐7 and MDA‐MB231 cells (5 × 10^5^ cells/well) were treated with TTP‐8 (0, 10, 20, and 30 μM) for 24 h. Whole‐cell extracts were prepared and Western blotting were performed with autophagy related antibodies. (D and E) MCF‐7 and MDA‐MB231 cells (5 × 10^5^ cells/well) were treated with TTP‐8 (30 μM) for 0, 12, 24, and 36 h. Whole‐cell extracts were prepared and Western blotting were performed with autophagy related antibodies. β‐Actin was used as a loading control. All the experiments were individually repeated at least three times.

The chemical structure of TTP‐8 is depicted in Figure 1D. In MCF‐7 and MDA‐MB231 cells, TTP‐8 was added for 24 h at the specified concentrations and then the cytotoxic effect was confirmed. We noted that TTP‐8 significantly suppressed cell viability in MCF‐7, MDA‐MB231 cells, while MCF‐10A cells showed a comparatively higher survival rate (Figure 2A). The induction of autophagic cell death by TTP‐8 was verified through the autophagy marker, LC3 protein, which showed a concentration‐dependent increase in LC3 II (Figure 2B). We also assessed whether TTP‐8 influences the expression of autophagy‐related proteins and the results demonstrated an upregulation of Atg7 and p‐Beclin‐1 protein expression (Figure 2C). Furthermore, a time‐dependent analysis of LC3 protein expression was conducted using Western blot after treatment with TTP‐8 at 0, 12, 24, and 36 h, confirming an increase in both LC3 and other autophagy‐associated proteins in BC cells (Figure 2D,E).

### 3.2 TTP‐8 induced autophagy in BC cells

**FIGURE 3:**
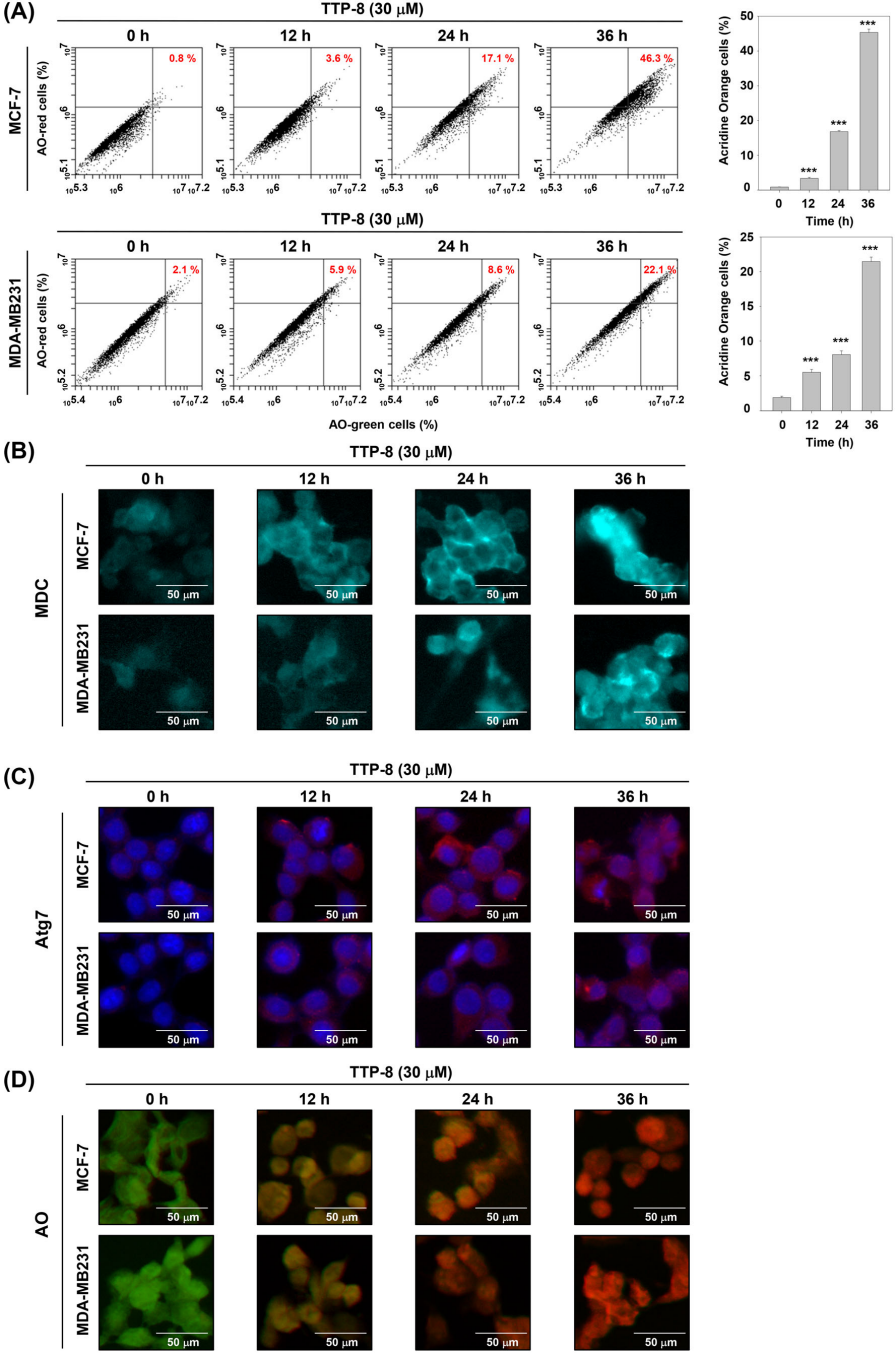
TTP‐8 induces autophagy. (A) MCF‐7 and MDA‐MB231 cells (5 × 10^5^ cells/well) were treated with TTP‐8 (30 μM) for 0, 12, 24, and 36 h. The cells treated with TTP‐8 were then incubated with acridine orange (AO) at 37°C for 30 min and analyzed using flow cytometry. Data represent means ± SD. *p < .05, **p < .01, ***p < .001 versus non‐treated cells. (B) MCF‐7 and MDA‐MB231 cells (2 × 10^4^ cells/well) seeded on 8 well chambers and treated TTP‐8 (30 μM) for time intervals. Then cells were stained with monodansylcadaverine (MDC) solution for 30 min, and the formation of autophagic vacuoles were detected by confocal microscopy. The scale bar is 50 μm. (C) MCF‐7 and MDA‐MB231 cells (2 × 10^4^ cells/well) seeded on 8 well chambers and treated TTP‐8 (30 μM) for time intervals. Immunocytochemistry (ICC) was performed with Atg7 antibody and nuclei were detected through DAPI staining. The scale bar is 50 μm. (D) MCF‐7 and MDA‐MB231 cells (2 × 10^4^ cells/well) seeded on 8 well chambers and treated TTP‐8 (30 μM) for time intervals. AO staining assay was performed with confocal microcopy, and acid vesicular organelle (AVO) was dyed red. The scale bar is 50 μm. All the experiments were individually repeated at least three times.

Experiments were then conducted to investigate whether TTP‐8 causes autophagy. Using AO, a cell‐permeable dye that fluoresces in acidic vesicular organelles (AVOs), flow cytometry was utilized to detect autolysosomes associated with autophagy.^21^ An increase in cells stained with AO confirmed the induction of autophagy (Figure 3A). MDC staining revealed that the formation of acidic vesicular organelles also increased with longer durations of TTP‐8 treatment (Figure 3B). Furthermore, the expression of Atg7 was observed to be time‐dependent (Figure 3C). Confocal microscopy results with AO also displayed an increase in red fluorescence, similar to the observations in Figure 2A (Figure 3D).

### 3.3 Autophagy inhibitors attenuated TTP‐8 related cell death

**FIGURE 4:**
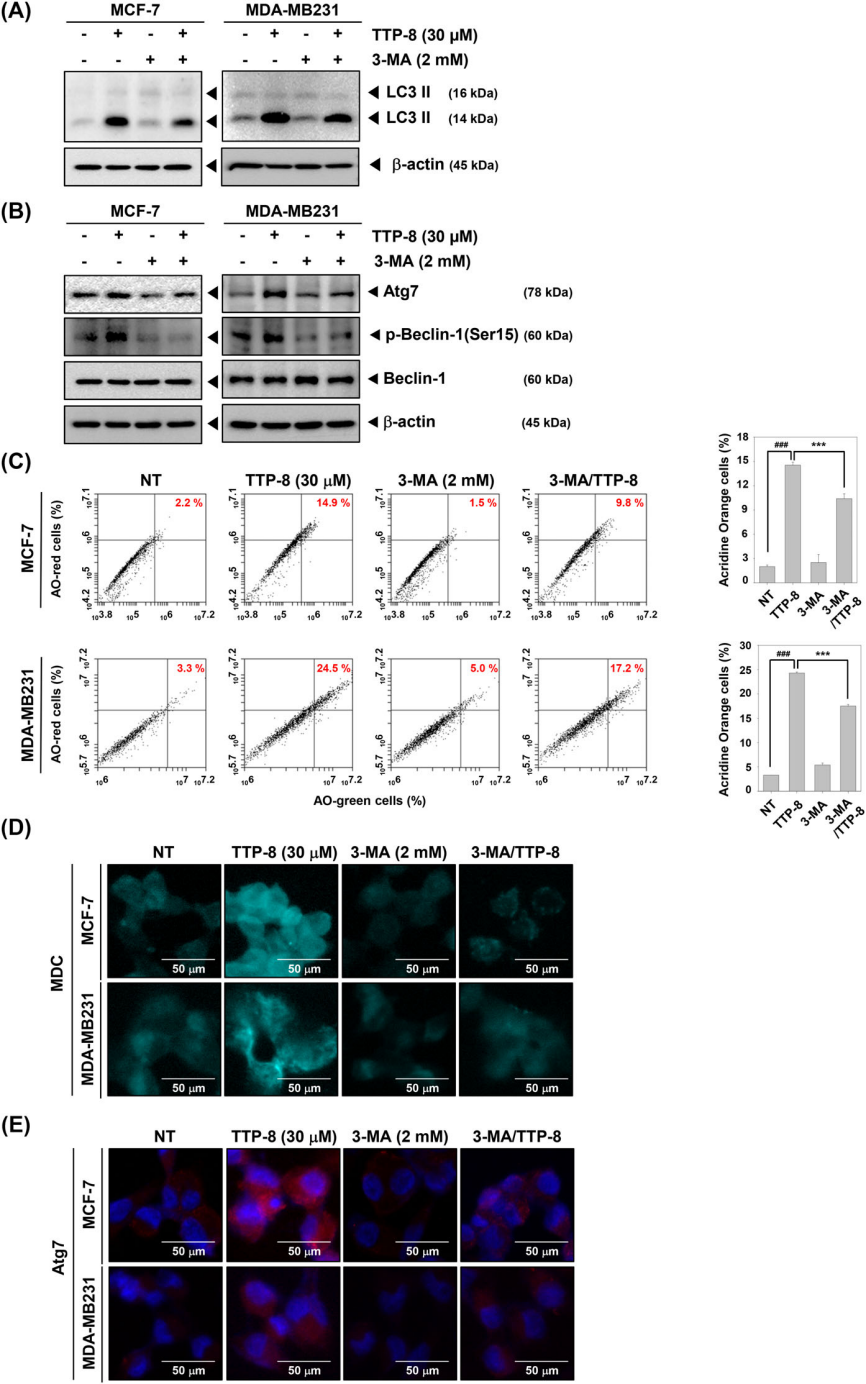
TTP‐8 restores autophagy inhibited by 3‐MA. (A and B) MCF‐7 and MDA‐MB231 cells (5 × 10^5^ cells/well) were treated with TTP‐8 (30 μM) and 3‐MA (2 mM) for 24 h. Whole‐cell extracts were prepared and Western blotting were performed with autophagy related antibodies. β‐Actin was used as a loading control. (C) MCF‐7 and MDA‐MB231 cells (5 × 10^5^ cells/well) were treated with TTP‐8 (30 μM) and 3‐MA (2 mM) for 24 h. The cells treated with TTP‐8 and 3‐MA were then incubated with acridine orange (AO) for 30 min at 37°C and analyzed using flow cytometry. Data represent means ± SD. #*p* < .05, ##*p* < .01, ###*p* < .001 versus non‐treated cells and *p < .05, **p < .01, ***p < .001 versus 3‐MA treated cells. (D) MCF‐7 and MDA‐MB231 cells (2 × 10^4^ cells/well) seeded on 8 well chambers. After that treated with TTP‐8 (30 μM) and 3‐MA (2 mM) for 24 h. The cells were stained with MDC solution for 30 min and detected by confocal microscopy. The scale bar is 50 μm. (E) MCF‐7 and MDA‐MB231 cells (2 × 10^4^ cells/well) seeded on 8 well chambers and treated with TTP‐8 (30 μM) and 3‐MA (2 mM) for 24 h. After that immunocytochemistry (ICC) was performed with Atg7 antibody. Nuclei were detected through DAPI staining. The scale bar is 50 μm. All the experiments were individually repeated at least three times.

Autophagy triggered by TTP‐8 was suppressed when cells were treated with the autophagy inhibitor, 3‐MA. Interestingly, LC3 expression, which was inhibited by 3‐MA, showed partial recovery when TTP‐8 was co‐administered with 3‐MA (Figure 4A). Similarly, the expression of Atg7 and p‐Beclin‐1 followed the trends shown in Figure 4A (Figure 4B). Flow cytometry results from the Acridine orange assay revealed that autophagy induced by TTP‐8 was mitigated by 3‐MA (Figure 4C). The presence of autophagic vacuoles and Atg7 expression, as depicted in Figure 4D,E respectively, were consistent with the previous data. These results demonstrate that autophagy induced by TTP‐8 can be reversed by autophagy inhibitor (3‐MA).

### 3.4 TTP‐8 regulated the activation of Wnt/β‐catenin signaling pathway in BC cells

**FIGURE 5:**
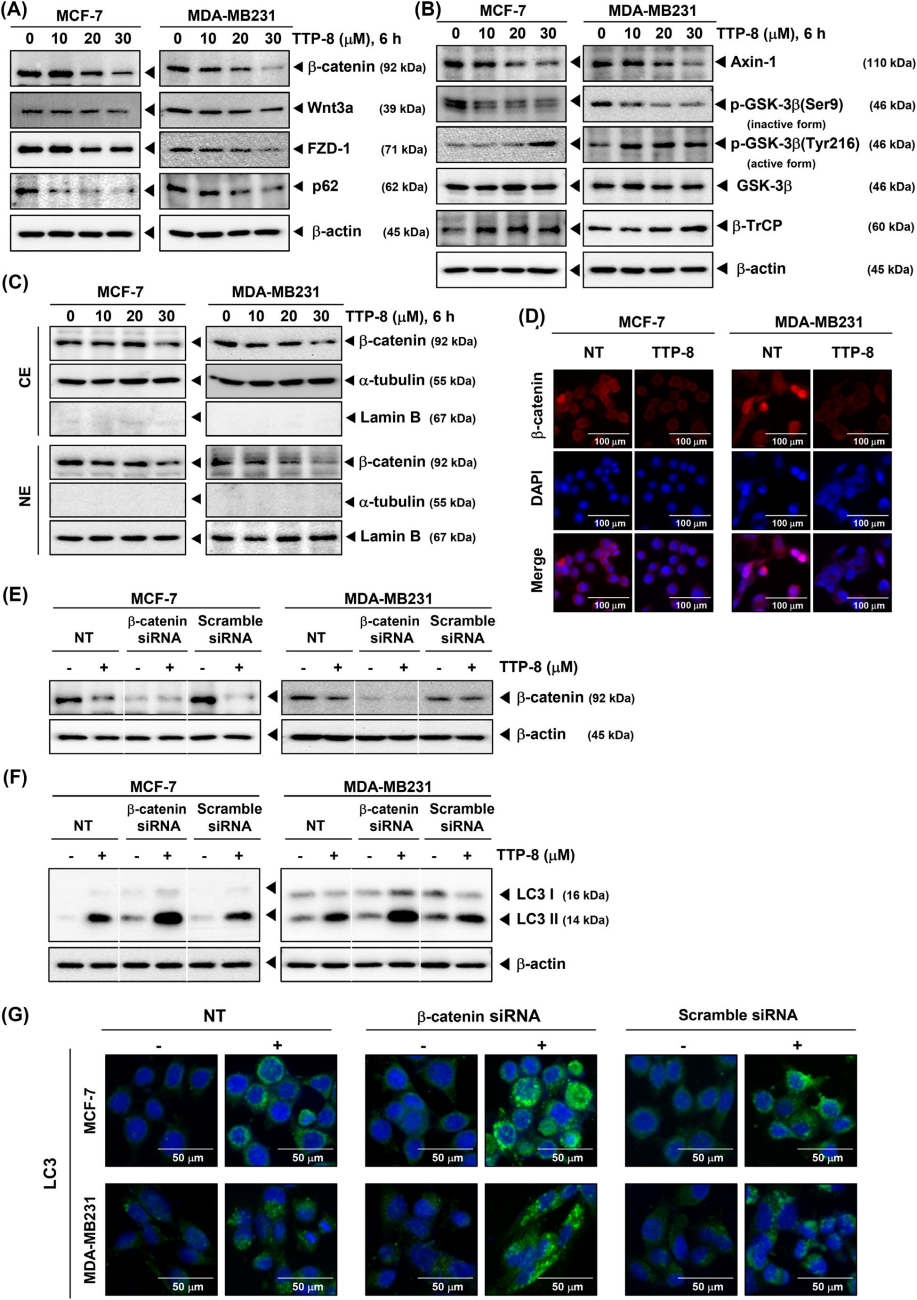
TTP‐8 induces autophagy via the Wnt/β‐catenin signaling pathway in BC cells. (A and B) MCF‐7 and MDA‐MB231 cells (5 × 10^5^ cells/well) were treated with TTP‐8 (0, 10, 20, and 30 μM) for 6 h. Whole‐cell extracts were prepared and Western blotting were performed. (C) MCF‐7 and MDA‐MB231 cells (5 × 10^5^ cells/well) were treated with TTP‐8 (0, 10, 20, and 30 μM) for 6 h. Then, cytoplasmic extracts (CE) and nuclear extracts (NE) were prepared. Western blotting was performed, α‐tubulin or Lamin B was used as a loading control for cytoplasmic or nuclear extracts. (D) MCF‐7 and MDA‐MB231 cells (2 × 10^4^ cells/well) seeded on 8 well chamber. ICC was performed after treatment of TTP‐8 (30 μM) for 6 h. Nuclei were stained with DAPI and detected with confocal microscopy. The scale bar is 100 μm. (E and F) MCF‐7 and MDA‐MB231 cells (5 × 10^5^ cells/well) were transfected with β‐catenin siRNA and scrambled siRNA (50 nM) for 24 h using Neon™ Transfection System. After transfection, TTP‐8 (30 μM) was treated for 6 or 24 h, followed by Western blotting with whole cell lysate. (G) MCF‐7 and MDA‐MB231 cells were transfected with β‐catenin siRNA and scrambled siRNA (50 nM) for 24 h using Neon™ Transfection System. Transfected cells (2 × 10^4^ cells/well) were seeded in 8 well chambers then followed by treatment of TTP‐8 (30 μM) for 24 h. Thereafter, ICC was performed using LC3 antibody. Nuclei were stained with DAPI and detected with confocal microscopy. The scale bar is 50 μm. All the experiments were individually repeated at least three times.

It has been reported that β‐catenin impedes autophagy by inhibiting LC3 activation.^15^ We investigated whether TTP‐8 impacts the Wnt/β‐catenin signaling pathway in BC cells. Our findings indicate that TTP‐8 suppresses the expression of β‐catenin, Wnt3a, FZD‐1, Axin‐1, and p‐GSK‐3β in a concentration‐dependent manner. Moreover, levels of p62, a substrate of autophagy used as an indicator of autophagy activity, were also reduced (Figure 5A,B). Further analysis revealed that β‐catenin expression was diminished in both the cytoplasmic and nuclear compartments (Figure 5C). ICC performed with confocal microscopy also confirmed the nuclear translocation of β‐catenin (Figure 5D).

### 3.5 The silence of β‐catenin triggered a stronger autophagy

Based on the data in Figure 5A, the activity of β‐catenin was verified by silencing it using siRNA transfection (Figure 5E). To examine the role of β‐catenin in autophagy, we depleted β‐catenin and assessed LC3 expression, a marker of autophagy activation, through Western blot analysis. We observed that autophagy was more pronounced when β‐catenin was silenced (Figure 5F). LC3 puncta formation, a biological indicator of autophagy, increased following siRNA‐mediated silencing of β‐catenin, as demonstrated using confocal microscopy (Figure 5G).

### 3.6 In silico interaction of TTP‐8 with β‐catenin

**FIGURE 6:**
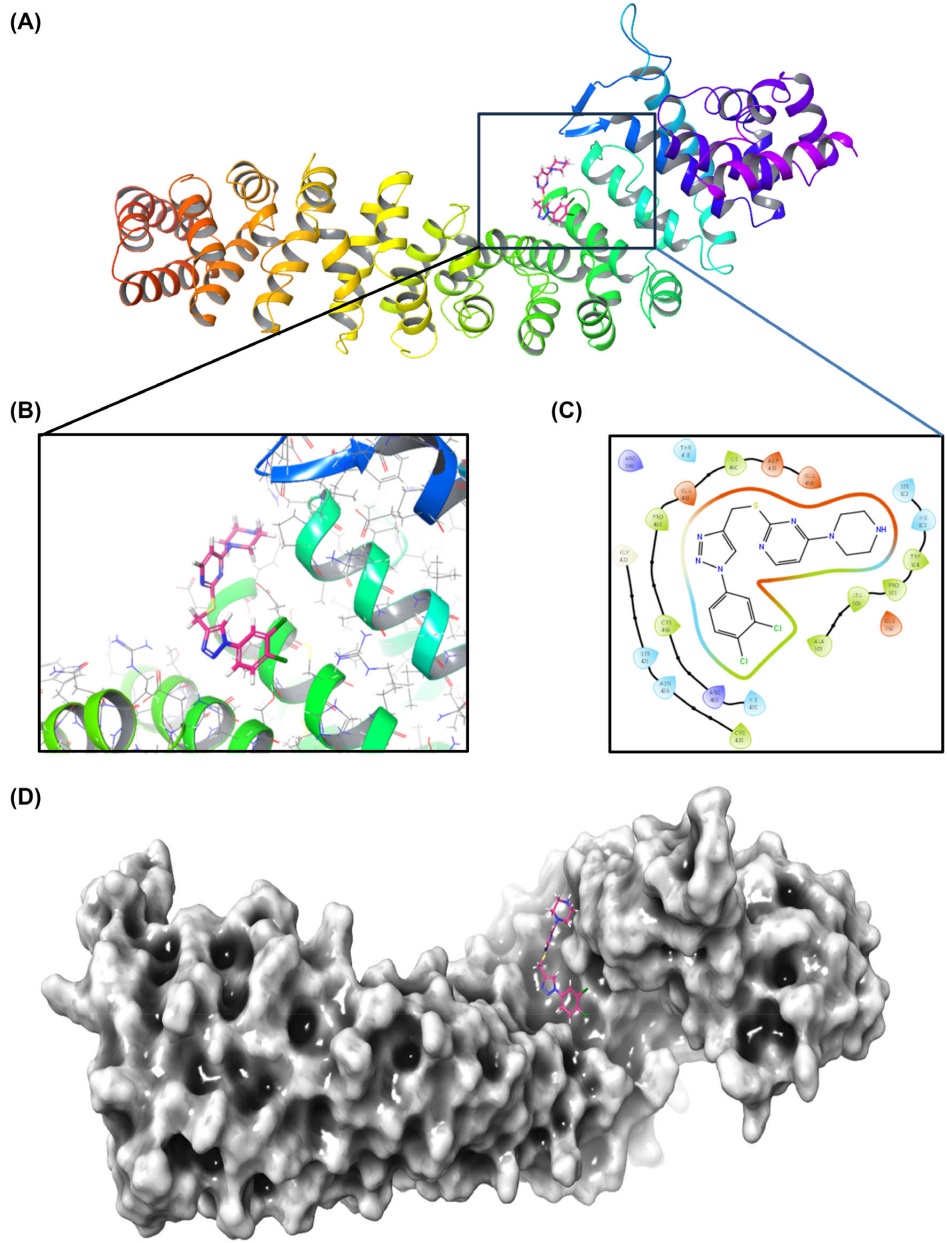
The representation of β‐catenin and TTP‐8 complex and binding pose of the TTP‐8 inside the binding pocket of β‐catenin. (A) Three dimension representation of TTP‐8 and β‐catenin complex. (B) Zoomed view of the binding mode of TTP‐8 in the binding pocket of the extended region of the XTcf3‐CBD. (C) 2D interaction diagram depicting all the key interaction of ligand with residues of β‐catenin. (D) Surface representation of β‐catenin and the binding mode of TTP‐8 in the binding domain of the extended region of the XTcf3‐CBD within the β‐catenin.

To assess the inhibitory effects of TTP‐8 on β‐catenin, molecular docking studies were conducted using AutoDock 4.2. These simulations investigated the interaction between TTP‐8 and the β‐catenin binding sites, specifically focusing on the XTcf3‐CBD, a domain integral to the binding of Tcf3‐CBD and variably involved with adenomatous polyposis coli, cadherin, and Axin. The simulations revealed that TTP‐8 bound to the extended region of the XTcf3‐CBD with a computed binding energy of −6.54 kcal/mol. Notable interactions were observed between TTP‐8 and key residues within the active site, including significant hydrophobic interactions with residues PRO463, ILE460, CYS466, TRP504, PRO505, LEU506, and ALA509, thereby enhancing the stability of the molecule within the binding pocket of β‐catenin (Figure 6A‐D).^38^

## 4 DISCUSSION

The objective of this study was to assess the anticancer properties of TTP‐8 in BC cells. BC remains the most prevalent cancer among women, and despite numerous available treatments, the discovery of new therapies remains critical due to the common issues of drug resistance and recurrence in cancer treatment.^39^, ^40^, ^41^ The relationship between cancer and autophagy has been well‐documented in several studies.^15^, ^42^, ^43^ Therefore, initially, we evaluated the cytotoxic effects of TTP‐8 on BC cells, subsequently confirming its involvement in inducing autophagy. The drug treatment concentrations were determined by IC50 values. To verify autophagy‐dependent cell death, we performed Western blot analyses with autophagy‐related proteins and employed various staining techniques to observe autophagosomes and autolysosomes, confirming the induction of autophagy.

Autophagy is a self‐degradative process that removes misfolded proteins, damaged organisms, and intracellular pathogens.^44^, ^45^ The role of autophagy as an important regulator in tumor development and treatment is drawing attention. Because LC3 I is converted to LC3 II during autophagy and binds to the membrane of autophagosome, LC3 is a direct marker of autophagosome. Furthermore, we confirmed the upregulation of Atg7 and p‐beclin‐1, as they are involved in the conversion of LC3.^30^, ^46^, ^47^ In our study, autophagy was stimulated by TTP‐8 to confirm the expression of related proteins in a concentration‐ and time‐dependent manner. The expression of P62, an autophagic flux marker, was also reduced.^31^, ^48^ Also, autophagosomes and autolysosomes formation were observed through AO staining and MDC staining. We noted that TTP‐8 restored autophagy activity inhibited by 3‐MA pre‐treatment. These results indicate that TTP‐8 has an anti‐tumor effect by inducing autophagy in BC cells.

The activation of the Wnt/β‐catenin signaling pathway is commonly observed in tumors, and it is the subject of extensive research.^49^, ^50^, ^51^ This pathway is initiated when the Wnt protein binds to the Frizzled/LRP6/LRP5 receptor complex, leading to the inhibition of GSK3β activity and the downregulation of Axin, which ultimately prevents the degradation of β‐catenin in the cytoplasm. Subsequently, accumulated β‐catenin translocates into the nucleus and enhances the expression of target genes.^52^, ^53^, ^54^ Our study found that TTP‐8 modulates the expression of proteins related to the Wnt/β‐catenin cascade in BC cells in a time‐dependent manner. We observed that TTP‐8 inhibited the nuclear translocation of β‐catenin. Additionally, the expression of LC3 was significantly increased by TTP‐8 in β‐catenin‐silenced MCF‐7 and MDA‐MB231 cells. These findings underscore the interconnection between Wnt/β‐catenin signaling and autophagy. However, several limitations in this study could impact the interpretation of the findings. Firstly, the investigation was conducted solely using in vitro cell lines (MCF‐7 and MDA‐MB231), which, while useful for initial screening, do not completely mimic the complex microenvironment and interactive cellular dynamics present within actual tumors. Another significant limitation is the absence of in vivo data using orthotopic and patient‐derived xenograft (PDX) models, which are critical for evaluating the clinical relevance of our findings. These models could provide a more accurate representation of how TTP‐8 behaves in a complex biological environment, including its bioavailability, pharmacokinetics, and interaction with the immune system. In vivo studies could also help in identifying any potential toxicity or adverse effects of TTP‐8, offering insights that are impossible to gain through in vitro experiments alone. As such, future research should aim to incorporate these models to validate the efficacy and safety of TTP‐8 as a potential treatment for BC. Another major limitation of this study is the lack of detailed molecular mechanisms underlying the action of TTP‐8. Although, we observed the upregulation of autophagy‐related proteins and suppression of the Wnt/β‐catenin signaling pathway, the specific interactions and pathways influenced by TTP‐8 at the molecular level remain unclear. Thus, without a deeper understanding of these mechanisms, the potential side effects and the precise therapeutic window of TTP‐8 cannot be completely deciphered. Overall, while the study provides promising data on potential of TTP‐8 as a cancer therapeutic, these limitations highlight the need for further research to completely establish its efficacy and safety profile in more physiologically relevant models.

**FIGURE 7:**
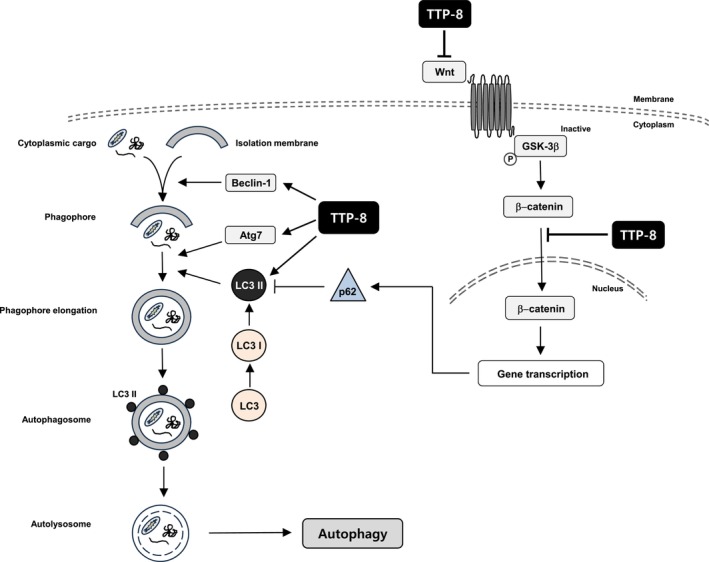
A schematic diagram illustrating the action mechanism of TTP‐8 in BC. The diagram above depicts the mechanism through which TTP‐8 can act in BC cells.

In conclusion, our study demonstrated that treatment with TTP‐8 led to increased levels of LC3, p‐Beclin‐1, and Atg7, which are key components of autophagy, in BC cell lines MCF‐7 and MDA‐MB231. This increase triggered the autophagic process, while concurrently inhibiting the Wnt/β‐catenin signaling pathway (Figure 7). Although further studies are needed to completely understand the mechanisms through which TTP‐8 operates, our findings indicate that TTP‐8 has potential as a therapeutic agent for promoting the death of BC cells.

## CONFLICT OF INTEREST STATEMENT

The authors declare no conflict of interest.
